# The Effects of Intraocular Pressure-Lowering Drops on the Tear Film Assessed by a Novel High-Resolution Tear Film Imager

**DOI:** 10.3390/diagnostics16101482

**Published:** 2026-05-13

**Authors:** Alice Verticchio Vercellin, Samuel Potash, Kira Manusis, Paul A. Sidoti, Richard B. Rosen, Brent A. Siesky, Keren Wood, Lily A. Greenberg, Peter D’Amelia, Edan Kenig, Norman J. Kleiman, David J. Brenner, George J. Eckert, Lucia Tanga, Carmela Carnevale, Masako Chen, David Qi, Minwoo Kwon, Gal Antman

**Affiliations:** 1 Ophthalmology, Icahn School of Medicine at Mount Sinai, New York, NY 10029, USA; samuel.potash@mssm.edu (S.P.); kmanusis@nyee.edu (K.M.); psidoti@nyee.edu (P.A.S.); rrosen@nyee.edu (R.B.R.); keren.woodshalem@mssm.edu (K.W.); masako.chen2@mountsinai.org (M.C.); minwoo.kwon@icahn.mssm.edu (M.K.); antmangal@gmail.com (G.A.); 2 Ophthalmology, New York Eye and Ear Infirmary of Mount Sinai, New York, NY 10003, USA; lilya.greenberg@mountsinai.org (L.A.G.); peter.damelia@mountsinai.org (P.D.); 3 AdOM Advanced Optical Technologies Ltd., Lod 71520, Israel; edan@adom-tech.com; 4 Environmental Health Sciences, Mailman School of Public Health, Columbia University, New York, NY 10032, USA; njk3@cumc.columbia.edu; 5 Center for Radiological Research, Columbia University Irving Medical Center, New York, NY 10032, USA; djb3@cumc.columbia.edu; 6 Department of Biostatistics and Health Data Science, Indiana University School of Medicine and Richard M. Fairbanks School of Public Health, Indianapolis, IN 46202, USA; geckert@iu.edu; 7 IRCCS-Fondazione Bietti, 00184 Rome, Italy; lucia.tanga@fondazionebietti.it (L.T.); carmela.carnevale@fondazionebietti.it (C.C.); 8 Faculty of Medicine, Tel Aviv University, Tel Aviv 69978, Israel

**Keywords:** tear film, tear film imager, IOP-lowering medications

## Abstract

**Background/Objectives:** The aim of this study was to investigate the effects of intraocular pressure (IOP)-lowering drops on the sublayers of the human tear film as assessed by a novel nanometer-resolution Tear Film Imager (TFI, AdOM, Israel). **Methods:** In a prospective, cross-sectional study, 98 eyes from 56 adult human subjects were imaged using the TFI. The dataset included data from 18 eyes from 12 subjects treated with preserved IOP-lowering drops and 80 eyes from 44 control subjects not under ocular hypotensive therapy. Subjects in the IOP treatment group used a variety of IOP-lowering medications, including prostaglandin analogs, beta-blockers, carbonic anhydrase inhibitors, alpha agonists, and combination drops. A linear mixed effects model was used to assess the association between IOP-lowering therapy and tear film (TF) metrics, controlling for age and intra-individual correlation. The following parameters were measured: muco-aqueous layer thickness (MALT), muco-aqueous layer thinning rate (MALTR), lipid layer thickness (LLT), lipid map uniformity (LMU), inter-blink intervals (IBI), and lipid break-up time (LBUT). **Results:** Average ages significantly differed (*p* = 0.013) between the treatment group (66.5 years) and control group (average age 51.5 years), and thus results were adjusted for age accordingly. IOP was 17.1 mmHg in the treatment group and 16.1 mmHg in the control group. When analyzing the sublayers of the TF, MALTR had a significant association with IOP-lowering therapy after adjusting for age, with a difference of −52.68 nm/s; 95% confidence interval [−96.87, −8.48]; *p*-value = 0.020. Additionally, IBI was significantly associated with IOP-lowering therapy after log transformation (*p* = 0.049), with shorter IBI in the treatment group. All other metrics (MALT, LLT, LMU, and LBUT) were statistically insignificant (*p* > 0.05). **Conclusions:** These pilot results suggest that IOP-lowering drops may accelerate thinning of the TF, specifically the muco-aqueous layer. Longitudinal studies with significantly larger samples are needed to specify the differential impact of various ocular hypotensive therapies on the human TF and the clinical implications of these findings.

## 1. Introduction

The tear film (TF) is a thin, multi-layered liquid covering the anterior corneal surface, composed of a deep muco-aqueous layer produced by the lacrimal glands and goblet cells, and a superficial lipid layer secreted by the meibomian glands [1,2]. As the outermost optical surface of the eye, the TF is essential for visual function, providing a smooth surface that smooths micro-irregularities in the underlying anterior epithelial surface and reduces higher-order optical aberrations, improving contrast sensitivity and visual acuity. In addition, the TF provides ocular surface lubrication and antimicrobial defense [3,4,5]. Disruption of TF stability can lead to dry eye disease (DED), a chronic condition characterized by tear-film instability, hyperosmolarity, and ocular discomfort [6,7].

Traditional methods for evaluating the TF, including fluorescein tear break-up time, Schirmer testing, and ocular surface staining, are widely used clinically but are limited by their subjective, qualitative nature and inability to assess individual TF sublayers independently [8]. More recently, TF imaging devices capable of providing a quantitative assessment of the TF have been developed, including the Oculus Keratograph 5M, the LipiView interferometer, and the Ocular Surface Analyzer [9,10,11]. The Tear Film Imager (TFI; AdOM Advanced Optical Technologies, Israel) represents an advancement over these technologies, as the only device that provides nanometer-resolution, time-resolved hyperspectral interference imaging of tear film sublayers, enabling quantification of both interblink tear-film dynamics and derived summary biomarkers [12,13]. The TFI quantifies key parameters including muco-aqueous layer thickness (MALT), muco-aqueous layer thinning rate (MALTR), lipid layer thickness (LLT), lipid break-up time (LBUT), inter-blink interval (IBI), and lipid map uniformity (LMU), providing a comprehensive, non-invasive assessment of TF health [12,13,14,15,16].

Topical intraocular pressure (IOP)-lowering medications, including prostaglandin analogs (PGAs), beta-blockers, carbonic anhydrase inhibitors (CAIs), alpha-agonists, and Rho kinase inhibitors [17,18], are the mainstay of therapy for glaucoma, the leading cause of irreversible blindness worldwide, and ocular hypertension (OHT). While effective, these medications carry a significant iatrogenic ocular surface burden, with ocular surface disease (OSD) prevalence considerably higher in treated glaucoma patients compared to untreated controls [19,20]. A principal driver of this toxicity is benzalkonium chloride (BAK), a preservative that disrupts the TF, reduces goblet cell density, and activates inflammatory pathways at the ocular surface [21,22,23,24,25,26]. Beyond BAK, the active drug components may independently contribute to ocular surface injury [27,28,29]. Prior studies using various TF imaging devices have documented reduced lipid layer thickness, increased meibomian gland dropout, and worsened tear film stability in glaucoma patients on topical therapy compared to controls [30,31,32,33,34,35,36,37]. However, these studies have assessed only composite measures of TF parameters, and none have resolved the temporal dynamics of the MALT and LLT as independent sublayers, leaving the sublayer-level mechanisms of TF disruption induced by IOP-lowering medications entirely uncharacterized.

Our group previously published the first quantitative assessment of TF sublayer changes across the human lifespan in healthy adults using the TFI, demonstrating significant age-related changes in both MALT and LLT [16]. These normative data provide an essential reference framework against which pathologic or drug-induced changes in TF sublayers can be contextualized.

To date, no study has utilized the TFI or any device with comparable nanometer-resolution sublayer quantification capability to assess the specific effects of IOP-lowering medications on MALT and LLT independently. Identifying the specific sublayer changes associated with topical glaucoma therapy could clarify the mechanisms underlying drug-induced OSD and inform decisions about preservative systems. The ultimate goal is to minimize iatrogenic ocular surface disease without compromising IOP control. The objective of this study is therefore to investigate the effects of IOP-lowering eye drops on individual TF sublayers compared to age-adjusted healthy controls.

## 2. Materials and Methods

A total of 98 eyes from 56 adult subjects were evaluated in a prospective, cross-sectional pilot study conducted at The New York Eye and Ear Infirmary of Mount Sinai, New York, NY. Written informed consent was obtained from all participants prior to participation. This study was conducted in accordance with the Declaration of Helsinki and was approved by the Institutional Review Board at the Icahn School of Medicine at Mount Sinai, New York, NY, USA (STUDY-22-01415).

The study included 18 eyes from 12 subjects treated with preserved IOP-lowering drops and 80 eyes from 44 control subjects not under ocular hypotensive therapy. Inclusion criteria for the control group included patients aged 18 years or older with no history of ocular disease or ophthalmic surgical or medical treatments. Study participants in the control group were excluded if they reported ocular symptoms, were treated with any ocular medication (including artificial tears), had undergone any prior eye surgery, had any systemic disease that could affect TF (e.g., Sjogren disease), or wore contact lenses. The IOP-lowering medications group included patients with primary open-angle glaucoma (as diagnosed by a fellowship-trained glaucoma specialist based on a gonioscopically open angle and structural and functional optic nerve damage consistent with glaucoma) and ocular hypertension (baseline IOP > 21 mmHg in the absence of structural or functional optic nerve damage). Exclusion criteria included: refractive error > +9 Diopters and <–9 Diopters in spherical equivalent; severe glaucoma (legally blind or near blind); use of contact lenses; history of intraocular surgery within 6 months; eye disease other than glaucoma; use of ocular medications (other than IOP-lowering medications for glaucoma or eye lubricant); use of ocular medications 3 h before the study research visit; neurological disease; psychosis or other diseases that could prevent reliable eye exams; and severe, unstable, or uncontrolled cardiovascular, renal, or pulmonary disease.

**Figure 1:**
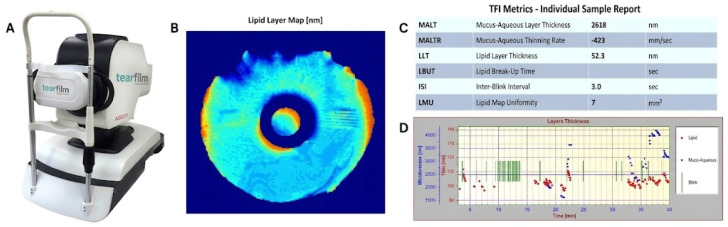
(**A**) Tear Film Imager (TFI) device and (**B**–**D**) representative TFI test report including lipid layer map, tear film metrics, and layers thickness, respectively.

All study participants’ ocular health was evaluated comprehensively by a single cornea specialist. All study subjects completed the Ocular Surface Disease Index (OSDI) questionnaire, a 12-question validated questionnaire used to assess dry eye symptoms [38]. Participants then underwent a non-contact assessment of their TF using the novel, non-invasive TFI (software version 3.11, AdOM, Lod, Israel), which uses spectral interference to image TF sublayers with nanometer resolution [12]. Briefly, the TFI provides a 40 s non-invasive quantitative assessment of the derived summary biomarkers, including the MALT, LLT, IBI, and LMU (Figure 1). Room temperature and humidity were recorded in testing rooms using a combined thermometer and hygrometer (Govee Hygrometer Thermometer, model H5075, Shenzhen Intellirocks Tech. Co., Ltd., Shenzhen, Guangdong, China). TFI measurements were captured by one of three trained operators. The TFI exams were done prior to any other exam or utilization of drops. The subjects in the IOP-lowering group reported not installing any drops for at least one hour prior to the exam. Only high-quality measurements, as demonstrated by quality parameters (test duration (overall recording time of valid data) > 6 s; position score (the deviation of the eye from the required position) < 21; data continuity score (the longest sequence of valid data recording) > 2.5 s) were included for analysis.

Statistical calculations were performed using Python (v3.12.13). A linear mixed effects model was used to assess the association between IOP-lowering therapy and TF metrics, controlling for age and intra-individual correlation. Due to right-skewed distributions, MALT, LLT, IBI, LMU, and LBUT were natural log-transformed prior to analysis. MALTR, which contains negative values, was retained on the raw scale. A two-sided 5% significance level was used for all tests.

## 3. Results

### 3.1. Participant Demographics

A total of 98 eyes from 56 subjects were included in this study, comprising 18 eyes from 12 subjects in the IOP-lowering therapy group and 80 eyes from 44 subjects in the control group. Table 1 illustrates the demographics of this population. The IOP-lowering therapy group included 9 subjects diagnosed with POAG, and 3 with ocular hypertension. Subjects used a variety of IOP-lowering medications, including prostaglandin analogs, beta-blockers, carbonic anhydrase inhibitors, alpha agonists, and combination drops. The majority (11/12) were treated exclusively with BAK-preserved formulations; one subject was also using a preservative-free prostaglandin analog (tafluprost) in addition to BAK-containing drops. The mean age of the IOP-lowering therapy group was 66.5 ± 11.5 years, which was significantly older than the control group (51.5 ± 19.4 years; *p* = 0.013), and thus the results were adjusted for age. There were no statistically significant differences between groups in gender distribution (IOP-lowering group: 4 female, 8 male; control group: 18 female, 26 male; *p* = 0.634) or racial/ethnic composition (*p* = 0.354). Mean IOP was 17.1 ± 5.1 mmHg in the IOP-lowering therapy group and 16.1 ± 2.9 mmHg in the control group, with no statistically significant difference between groups (*p* = 0.342). OSDI scores were comparable between the IOP-lowering therapy group (13.1 ± 17.1) and the control group (7.9 ± 12.1; *p* = 0.246).

### 3.2. TFI Biomarkers

**Figure 2:**
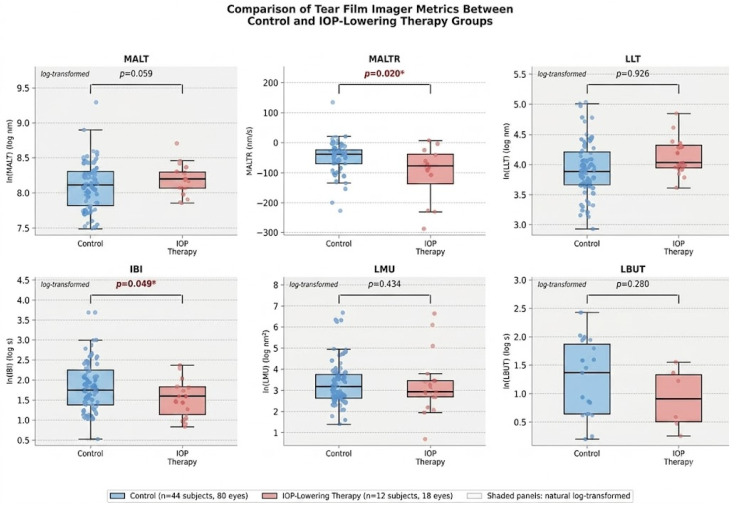
Comparison of Tear Film Imager metrics between the control and IOP-lowering therapy groups. Box plots display the median (horizontal line), interquartile range (box), and 1.5x interquartile range (whiskers) for each metric. Individual data points are overlaid with jitter. *p*-values were derived from a linear mixed effects model adjusting for age and intra-individual correlation. MALT, LLT, IBI, LMU, and LBUT were natural log-transformed prior to analysis. IBI: inter-blink interval; IOP: intraocular pressure; LBUT: lipid break-up time; LLT: lipid layer thickness; LMU: lipid map uniformity; MALT: muco-aqueous layer thickness; MALTR: mu-co-aqueous layer thinning rate. * *p* < 0.05.

When analyzing the individual sublayers of the tear film, MALTR had a statistically significant association with IOP-lowering therapy after adjusting for age, with a mean of −101.5 ± 95.6 nm/s in the IOP-lowering group compared to −49.8 ± 56.3 nm/s in the control group (difference = −52.68, 95% CI [−96.87, −8.48]; *p* = 0.020). Additionally, IBI showed a statistically significant association with IOP-lowering therapy after log transformation, with a mean of 5.17 ± 2.41 s in the IOP-lowering group compared to 7.82 ± 6.63 s in the control group (effect size = −0.381 on the log scale, 95% CI [−0.760, −0.001]; *p* = 0.049). The remaining TFI biomarkers (MALT, LLT, LMU, and LBUT) did not reach statistical significance after adjusting for age (*p* > 0.05) (Table 2 & Figure 2). With respect to image quality parameters, no significant differences were observed between groups in test duration, signal score, position score, or data continuity score (all *p* > 0.05) (Table 2).

## 4. Discussion

This pilot study is the first to quantitatively assess the effects of IOP-lowering drops on individual TF sublayers at nanometer resolution using the TFI. Our principal finding is that IOP-lowering therapy was significantly associated with an accelerated MALTR after adjusting for age (Table 2 & Figure 2), suggesting that chronic topical therapy with IOP-lowering medications may destabilize the dynamic properties of the muco-aqueous TF sublayer. MALTR reflects the rate at which the muco-aqueous layer thins between blinks and is therefore a sensitive marker of dynamic TF stability that captures aqueous evaporation and mucin layer destabilization in real time [12,13]. Notably, our group previously demonstrated that MALTR was not significantly associated with age in healthy adults [16], whereas MALT decreased significantly with age in that same population (*p* = 0.164 and *p* = 0.024, respectively). The significant association between IOP-lowering therapy and MALTR in the present study, therefore, suggests that the accelerated MALTR observed here reflects drug-induced TF disruption rather than age-related physiological change, lending further support to the specificity of this finding. IBI was also significantly associated with IOP-lowering therapy after log transformation, with shorter intervals observed in the treatment group, suggesting a possible increase in blink frequency in patients on chronic topical therapy. Notably, IOP was comparable between the IOP-lowering therapy and control groups (Table 1), suggesting that IOP itself is unlikely to be a confounder of the observed TF differences. Furthermore, OSDI scores were not significantly different between groups (Table 1), suggesting that the accelerated MALTR observed in the IOP-lowering therapy group may represent subclinical TF disruption not yet manifesting as symptomatic dry eye disease, analogous to the asymptomatic TF sublayer changes detected by the TFI in healthy aging adults in our prior study [16].

The observed acceleration in MALTR in the treatment group is consistent with the broader literature documenting significant ocular surface toxicity associated with chronic topical IOP-lowering medications. The prevalence of ocular surface disease in glaucoma patients has been reported to range from 38.5–75%, compared to 21.7–35% in untreated controls, highlighting the substantial iatrogenic ocular surface burden of these medications [28]. The principal driver of this toxicity is BAK, the most widely used preservative in topical ophthalmic formulations, which disrupts the TF lipid layer, reduces goblet cell density, and activates corneal and conjunctival inflammatory pathways, manifesting clinically as reduced TBUT, worsened Schirmer scores, corneal staining, and dry eye symptoms [21,22,23,24,25,26]. The connection between BAK-mediated goblet cell loss and accelerated MALTR is particularly relevant. As goblet cells are the primary source of the mucins that stabilize the muco-aqueous layer, their loss would be expected to reduce mucin availability and compromise muco-aqueous layer integrity, potentially contributing to the accelerated muco-aqueous thinning rate observed in our study [19,21,39,40]. Beyond BAK, the active drug components themselves may contribute independently to ocular surface injury; PGAs have been associated with meibomian gland dysfunction and periocular adnexal changes including hypertrichosis and periorbital skin changes, while beta-blockers have been shown to reduce basal tear turnover and alter tear film mucus composition independently of preservative effects, and rho kinase inhibitors have been implicated in conjunctival hyperemia, cornea verticillata, blepharitis, and corneal epithelial changes [41,42,43]. The accelerated MALTR observed in our study may therefore reflect the cumulative effects of both BAK-mediated goblet cell loss and active drug-induced alterations in glandular function and TF dynamics.

Prior studies using quantitative TF imaging devices have consistently demonstrated TF disruption in glaucoma patients on topical therapy, though none have dynamically assessed MALT as an independent sublayer at nanometer resolution. Studies utilizing LipiView interferometry have demonstrated reduced LLT in glaucoma patients on topical therapy compared to controls, with medication duration correlating with the degree of LLT reduction [30,31,37]. Notably, the treated eye has been shown to have lower LLT than the untreated fellow eye in unilateral glaucoma patients, and LLT has been observed to decrease significantly within the first six months of treatment initiation alongside worsening OSDI scores [30,31,37]. Studies using the Keratograph 5M have similarly documented significantly higher meibomian gland dropout rates, worsened TBUT, and greater corneal staining in treated glaucoma patients compared to controls, with evidence suggesting that PGAs may contribute to meibomian gland atrophy independently of BAK, and that BAK-containing drops alongside longer treatment duration independently predict worse ocular surface outcomes [32,33,34,35,36]. Comprehensive automated ocular surface analysis using the IDRA Ocular Surface Analyzer further confirmed significantly lower LLT, TBUT, and tear meniscus height alongside higher meibomian gland loss area in glaucoma patients on topical therapy versus controls [29]. Collectively, these studies demonstrate consistent evidence of TF disruption in treated glaucoma patients, particularly affecting the lipid layer. Our finding of accelerated MALTR adds a novel dimension to this body of literature by suggesting that IOP-lowering therapy also affects the dynamic properties of the muco-aqueous sublayer. The clinical relevance of MALTR as a sensitive dynamic marker is further supported by a recent study which demonstrated that MALTR, but not static MALT, correlated significantly with dry eye symptom scores and meibomian gland dysfunction (MGD) findings in patients with dry eye and MGD assessed using the TFI, establishing MALTR as a promising objective surrogate marker of tear film instability across disease contexts [39]. Importantly, the biological plausibility of TFI detecting topical drug-induced TF changes is further supported by our group’s prior work demonstrating that the TFI can detect short-term TF sublayer changes in response to artificial tear instillation [14], underscoring its sensitivity as a tool for capturing dynamic TF responses to topical ocular agents.

Shorter IBI, reflecting more frequent blinking in the treatment group, may represent a reflexive response to chronic ocular surface irritation induced by topical medications and their preservatives. Increased blink frequency has been described as a compensatory mechanism in response to ocular surface discomfort and TF instability, as more frequent blinking serves to redistribute and replenish the TF [44]. This interpretation is consistent with the broader literature documenting BAK-mediated conjunctival and corneal inflammation, as well as goblet cell loss, both of which are known to increase ocular surface sensitivity [21,22,23,24,25,26]. Notably, OSDI scores were not significantly different between groups, suggesting that the increased blink frequency observed here may represent a subclinical adaptive mechanism occurring prior to the onset of symptomatic dry eye disease. This parallels our finding of accelerated MALTR—both may reflect early, pre-symptomatic TF disruption that the TFI is uniquely sensitive to detect. Future studies should examine whether IBI normalizes following transition to preservative-free formulations or after treatment of ocular surface disease.

While differences in MALT, LLT, LMU, and LBUT between groups did not reach statistical significance in this pilot study, their mean differences warrant careful interpretation in the context of limited statistical power. Although mean LMU was higher and LBUT lower in the treatment versus control groups (consistent with prior literature documenting lipid layer disruption and reduced tear stability in glaucoma patients on topical therapy [17,30,37]), the difference between groups for both parameters did not reach statistical significance. For LMU, this trend is best appreciated on the log scale, where the treatment group showed lower values than controls, as the skewed raw distribution is driven by outliers that obscure the central tendency. Mean LLT was marginally higher in the treatment group, contrary to prior LipiView studies [30,31,37]; however, the small difference did not reach statistical significance. The discrepancy between the current study and prior LipiView studies with respect to LLT may reflect differences in methodology, as the TFI measures LLT using hyperspectral interference imaging at nanometer resolution, which may capture different aspects of lipid layer physiology than interferometry-based devices. Additionally, the relatively small sample size of the IOP-lowering therapy group (12 subjects, 18 eyes) and the cross-sectional design of this pilot study may have limited our statistical power to detect differences in static TF parameters. Mean MALT was higher in the treatment group with a *p*-value approaching significance (*p* = 0.059), but this difference was in the opposite direction from what would be expected given that BAK-mediated goblet cell loss has been shown to reduce mucin availability and deplete the muco-aqueous layer [17,18,19]. One potential explanation is a paradoxical compensatory mucin hypersecretion, whereby BAK-preserved drops may trigger MUC5AC (the primary gel-forming mucin secreted by conjunctival goblet cells) dispersal from goblet cells even as they reduce goblet cell viability, as demonstrated in vitro [45,46,47,48]. This paradoxical hypersecretion could transiently increase MALT while simultaneously destabilizing its dynamic properties, consistent with the accelerated MALTR observed in this study. This concept remains hypothetical, but it could explain the observed trend toward higher MALT in the treatment group by reflecting a transient increase in mucin availability despite underlying disruption of goblet cell function and tear film stability. Importantly, this proposed mechanism is speculative and requires further validation in future experimental and clinical studies.

The accelerated MALTR observed in the IOP-lowering therapy group raises important questions about the relative contributions of preservatives versus active drug components to muco-aqueous layer instability. While our study did not directly compare these formulations, the established role of BAK in goblet cell toxicity and muco-aqueous layer disruption raises the hypothesis that preservative-free alternatives may partially mitigate the accelerated MALTR observed here, though this remains speculative given evidence that active drug components may contribute independently of preservative effects [21,22,23,24,25,26]. Notably, a prior study using the IDRA Ocular Surface Analyzer found no significant differences in TF parameters between preserved and preservative-free formulations, implicating the active drug itself as an independent contributor to TF disruption [29]. This highlights the importance of considering both the preservative system and the active drug component when evaluating the ocular surface impact of IOP-lowering therapy. These findings also highlight the potential utility of the TFI as a sensitive clinical tool for monitoring TF health in glaucoma or ocular hypertensive patients on topical therapy, enabling early detection of subclinical muco-aqueous layer instability before the onset of symptomatic dry eye disease, and potentially informing treatment decisions aimed at minimizing iatrogenic ocular surface disease while maintaining effective IOP control. Similarly, the significant association between IOP-lowering therapy and IBI suggests that the TFI may also capture compensatory blink behavior in response to drug-induced ocular surface irritation, providing a more complete picture of the functional impact of topical therapy on the ocular surface.

This study has several limitations. First, the relatively small sample size of the IOP-lowering therapy group (12 subjects, 18 eyes) limits statistical power and the generalizability of our findings. In this context, it is important to emphasize that, because of the insufficient statistical power, the absence of statistically significant differences in biomarkers such as MALT, LLT, LMU, and LBUT between controls and IOP-lowering groups should not necessarily be interpreted as evidence of no difference between the groups. Second, the cross-sectional design precludes assessment of the longitudinal effects of IOP-lowering therapy on TF sublayers or the impact of treatment duration on MALTR. Third, the IOP-lowering therapy group used a variety of medications including PGAs, beta-blockers, CAIs, alpha agonists, and combination drops, which prevented analysis of the differential effects of individual drug classes on TF sublayers. We further acknowledge that the IOP-lowering medications included in this study have different mechanisms of action and may affect the ocular surface through distinct pathways. In this pilot study, all treated subjects were pooled into a single treatment group for analysis, representing a major limitation because it may obscure differential effects among medication classes. Future studies with larger sample sizes may help clarify the specific effects of different classes of IOP-lowering medications on the ocular surface. Fourth, the IOP-lowering therapy group was significantly older than the control group, and despite age adjustment in our statistical model, residual confounding by age cannot be entirely excluded. Therefore, it is important to emphasize that future studies should include an appropriately age-matched control group. It is also important to acknowledge that participants in the IOP-lowering medication group were allowed to have a history of ocular lubricant use. To minimize the potential confounding effect of lubricants on the TF, subjects were instructed not to use any eye drops, including both ocular lubricants and IOP-lowering medications, within 3 h prior to each study visit. Participants who had used any eye drops within this 3-h window were excluded from the study visit. While the majority of subjects were on BAK-preserved formulations (11/12), the heterogeneity of drug classes and the absence of a preservative-free comparison group limit conclusions about the relative contributions of BAK versus active drug components to the observed MALTR acceleration. Also, in our study, we did not collect information regarding the duration of treatment with IOP-lowering medications in the treatment group. This represents a significant limitation, particularly given the known impact of treatment duration on the tear film. Longitudinal studies with larger, more diverse samples are needed to explore the cumulative impact of treatment duration and provide direct comparisons between individual drug classes and preservative-free formulations. This will further define the differential impact of IOP-lowering therapy on TF sublayers and clarify the clinical implications for managing the ocular surface in glaucoma or ocular hypertensive patients.

## 5. Conclusions

To the best of our knowledge this pilot study is the first to quantify the effects of IOP-lowering drops on individual TF sublayers at nanometer resolution. Our findings demonstrate a significant association between IOP-lowering therapy and accelerated MALTR, independent of static layer thickness. Increased IBI was also significantly associated with IOP-lowering therapy, with shorter intervals in the treatment group suggesting increased blink frequency as a possible compensatory response to chronic ocular surface irritation. Difference between treated and control groups for all other TF metrics did not reach statistical significance. These results suggest that chronic topical IOP-lowering medications may destabilize the dynamic properties of the muco-aqueous TF sublayer, with potential implications for ocular surface health in glaucoma and ocular hypertensive patients. Longitudinal studies with larger sample sizes are needed to further define the differential impact of individual ocular hypotensive agents and their preservative systems on the human TF sublayers and connect these findings to clinical endpoints and patient quality of life.

## Figures and Tables

**Table 1:** Demographics and ocular characteristics of the study participants. IOP: Intraocular pressure; OSDI: Ocular Surface Disease Index. * indicates statistical significance, *p* < 0.05.

Parameter	Control Group (*n* = 44 Subjects, 80 Eyes)	IOP-Lowering Group (*n* = 12 Subjects, 18 Eyes)	*p*-Value
**Age, years (mean ± SD)**	51.5 ± 19.4	66.5 ± 11.5	0.013 *
**Sex, Female/Male**	18/26	4/8	0.634
**Race/Ethnicity**	White: 18, Black: 6, Asian: 5, Hispanic: 5, Other: 10	White: 2, Black: 3, Asian: 3, Hispanic: 1, Other: 3	0.447
**IOP (mmHg, mean ± SD)**	16.1 ± 2.9	17.1 ± 5.1	0.342
**BCVA logMAR (mean ± SD)**	0.83 ± 0.32 (*n* = 76 eyes)	0.70 ± 0.22 (*n* = 17 eyes) (1 eye of 1 study subject had count fingers as BCVA)	0.077
**OSDI Score (mean ± SD)**	7.9 ± 12.1 (*n* = 43)	13.1 ± 17.1 (*n* = 11)	0.246

**Table 2:** Tear Film Imager biomarkers and image quality parameters (mean ± standard deviation) with number of subjects and eyes in parentheses, and linear mixed effects model results comparing the IOP-lowering therapy and control groups, including the adjusted mean difference between the IOP-lowering therapy and control groups after controlling for age and intra-individual correlation, with a 95% confidence interval and *p*-value. MALT, LLT, IBI, LMU, and LBUT were natural log-transformed prior to analysis; effect sizes for these metrics are reported on the log scale. Test duration, signal score, position score, and data continuity score are measures of image quality. IBI: inter-blink interval; IOP: intraocular pressure; LBUT: lipid break-up time; LLT: lipid layer thickness; LMU: lipid map uniformity; MALT: muco-aqueous layer thickness; MALTR: muco-aqueous layer thinning rate. ^†^ Effect size on log scale (natural log-transformed). * *p* < 0.05.

Metric	Control Group Mean ± SD (Subjects/Eyes)	IOP-Lowering Group Mean ± SD (Subjects/Eyes)	Difference	95% CI	*p*-Value
**TFI Biomarkers**					
**MALT (nm)**	3510.8 ± 1361.3 (43/78)	3732.7 ± 852.9 (12/18)	0.172 ^†^	[−0.007, 0.315]	0.059
**LMU (nm^2^)**	62.1 ± 134.4 (44/79)	95.9 ± 203.7 (12/17)	−0.249 ^†^	[−0.874, 0.374]	0.434
**LLT (nm)**	56.8 ± 29.1 (44/80)	65.4 ± 22.1 (12/18)	0.011 ^†^	[−0.213, 0.235]	0.926
**LBUT (s)**	4.3 ± 2.8 (15/19)	2.8 ± 1.4 (5/6)	−0.327 ^†^	[−0.943, 0.276]	0.298
**MALTR (nm/s)**	−49.8 ± 56.3 (36/58)	−101.5 ± 95.6 (8/12)	−52.68	[−96.87, −8.48]	**0.020** *
**IBI (s)**	7.8 ± 6.6 (43/80)	5.2 ± 2.4 (12/18)	−0.381 ^†^	[−0.760, −0.001]	**0.049**
**Measurement Quality Metrics**					
**Test Duration (s)**	18.1 ± 8.8 (44/80)	18.3 ± 7.9 (12/18)	1.27	[−3.75, 6.29]	0.614
**Position Score**	10.6 ± 5.9 (44/80)	7.4 ± 6.1 (12/18)	−3.23	[−6.73, 0.28]	0.071
**Data Continuity Score**	0.7 ± 1.2 (44/80)	2.4 ± 6.8 (12/18)	2.42	[−0.15, 4.99]	0.065

## Data Availability

The data presented in the study are included in the article; further inquiries can be directed to the corresponding author.
